# High-performance temperature regulation of nonlinear CSTRs via a hybrid stellar oscillation optimizer and differential evolution-based PID-F control

**DOI:** 10.1038/s41598-026-38354-5

**Published:** 2026-02-07

**Authors:** Serdar Ekinci, Cebrail Turkeri, Islam Gokalp, Yongliang Li, Dacheng Li

**Affiliations:** 1 Department of Computer Engineering, Bitlis Eren University, Bitlis, 13100 Turkey; 2 Department of Computer Engineering, Batman University, Batman, 72100 Turkey; 3 Department of Civil Engineering, Batman University, Batman, 72100 Turkey; 4 Birmingham Centre for Energy Storage, School of Chemical Engineering, University of Birmingham, Edgbaston, Birmingham, B15 2TT UK

**Keywords:** CSTR process, Stellar oscillation optimizer, Differential evolution, Hybridization, Mutation and crossover operators, Reactor temperature regulation, PID-F controller, Energy science and technology, Engineering, Mathematics and computing

## Abstract

This study introduces a hybrid stellar oscillation optimizer with differential evolution (hSOO-DE) for high-performance tuning of PID controllers with derivative filtering (PID-F) in nonlinear temperature regulation of continuous stirred tank reactors (CSTRs). The hybrid approach combines the global exploration capability of the stellar oscillation optimizer (SOO) with the local exploitation strength of differential evolution, ensuring a well-balanced search between diversification and intensification during parameter optimization. The proposed algorithm was applied to a benchmark nonlinear CSTR model and comprehensively compared with state-of-the-art metaheuristic optimizers SOO, birds of prey-based optimization (BPBO), covariance matrix adaptation evolution strategy (CMA-ES) and differential evolution (DE) as well as classical tuning techniques including Ziegler-Nichols, Tyreus-Luyben, and Simulink Tuner. The optimization objective jointly minimizes overshoot and integral absolute error to enhance transient and steady-state control quality. Statistical analyses, including boxplot evaluations and Mann-Whitney U-tests, demonstrate that hSOO-DE achieves the lowest mean objective value with minimal variance compared to recent optimizers. Time-domain results confirm superior transient performance, reflected in reduced rise and settling times and minimal overshoot, while integral performance indices verify improved steady-state precision. Validation against conventional PID-F tuning methods further highlights the robustness and reliability of the proposed design. The findings demonstrate that embedding DE within the oscillatory structure of the SOO yields a robust and efficient framework for PID-F controller tuning in nonlinear chemical reactor systems.

## Introduction

 Temperature regulation in nonlinear industrial processes remains one of the most challenging and practically significant problems in process systems engineering. As a key device widely utilized in the chemical^1,2^, pharmaceutical^3,4^, and energy^5,6^ industries, the continuous stirred tank reactor (CSTR) serves as a canonical benchmark that encapsulates strong nonlinearities, pronounced thermal-kinetic coupling, and potential multi-stability^7,8^. The safe and efficient operation of the CSTR critically depends on accurate, rapid, and robust temperature control, since modest deviations against the desired temperature can trigger undesirable side reaction, product degradation, or thermal runaway^9,10^. Consequently, the design of reliable and high-performance control strategies for CSTRs continues to attract sustained research attention from both academia and industry.

Classical control approaches such as on-off control and linear control have been applied in the regulation of the CSTR temperature^11–13^. Despite their simple implementation, these methods often perform inadequately in dealing with strong nonlinear environments of the CSTR and typically yield low control accuracy. To strengthen the capability to address nonlinearity, model-based control methodologies have been developed. For example, sliding mode control (SMC) has been adopted to enhance the temperature control of the CSTR by direct shaping of system dynamics through a defined sliding surface^14^. This method was further improved by integrating a generalized extended state observer and a genetic algorithm (GA) to handle additional matched disturbances. Model predictive control (MPC) has also been employed for CSTR temperature regulation which can offer high adaptability and good performance by discretizing the control process and using multiple linearization techniques^15^. In^16^, MPC was adopted to control the reactor temperature and achieved excellent control behavior with stabilized reactor operation, even under step changes in the feed flowrate^17^. compared the performance of MPC for temperature regulation with conventional controllers such as P, PI, PD, and PID. The results indicated that MPC achieved a relatively low percentage overshoot and good load disturbance rejection with a minimum settling time. An upgraded Adaptive MPC was later designed for controlling CSTR temperature in^18^, showing superior control performance to PID control, fuzzy control, and traditional MPC. Although the effectiveness of model-based control method has been validated, the difficulty in the development of accurate process modelling as well as the requirement for high computational cost limit its utilization for practical application, especially for large-scale systems. To overcome these limitations and provide efficient control solutions without reliance on detailed process models, control methods based on artificial intelligence (AI) have been explored and applied to regulate the temperature of the CSTR. In^19^, a model predictive controller based on deep learning neural network was developed and tuned using hybrid particle swarm optimization (PSO) and gravitational search algorithm (GSA) to control the temperature of a non-linear CSTR. In^20^, a soft actor-critic (SAC) reinforcement learning (RL) framework was employed for CSTR temperature control. Both methods outperformed conventional control approaches. Nevertheless, these AI-based controllers still rely heavily on large amounts of real data, incur significant computation cost, and require advanced domain expertise, leading to practical implementation of these advanced methodologies in industrial circumstances remain challenging.

The proportional-integral-derivative (PID) controller has long been regarded as standard in industrial practice for its structural simplicity and interpretability^21^. Therefore, numerous application cases can be found for their contribution in controlling the CSTR temperature^22^. However, conventional tuning method such as Ziegler-Nichols or Tyreus-Luyben techniques limited the adaptability of PID controller in nonlinear systems^23,24^. These tuning formulas are derived from linear approximations around a nominal operating point and thus fail to guarantee global optimality or robustness across the full nonlinear domain of operation. To address these shortcomings, advanced tuning techniques for PID controller have gained prominence over the past two decades^25^. demonstrated a PID controller tuned using fuzzy logic theory, and the simulation results for CSTR temperature control showed that it was more effective compared to the conventional PID to enhance stability of time domain performance of the reactor. Additionally, in^26^, a fuzzy-based PID controller exhibited superior setpoint tracking performance relative to the Ziegler-Nichols method.

In parallel, intelligent metaheuristic optimization algorithms have also emerged as powerful alternatives owing to their derivative-free nature and global search capabilities. Techniques inspired by swarm intelligence and evolutionary computation, such as PSO, GA, and differential evolution (DE), have been successfully applied to PID tuning in nonlinear process control. Performance indices such as integral absolute error (IAE), integral of squared error (ISE), or overshoot, are normally adopted to evaluate the formulating PID controllers thereby improving both transient and steady-state responses^27^. For CSTR temperature regulation, PSO-based PID tuning has been shown to outperform conventional tuning rules by minimizing the ISE criterion and enhancing closed-loop performance^28^. Comparative investigations using PSO, GA, and DE further demonstrated that metaheuristic-based tuning strategies can significantly improve controller performance relative to classical methods, although their effectiveness remains sensitive to algorithmic parameter settings and search dynamics^29,30^.

More recently, hybrid and advanced metaheuristic-based PID tuning strategies have been increasingly reported to enhance closed-loop performance in nonlinear and uncertainty-prone systems. For instance, a hybrid framework combining harmony search with dwarf mongoose optimization (HS-DMOA) was employed to improve the dynamic performance of a PID-controlled benchmark regulation problem^31^. In a related direction, a recent study adopted the artificial hummingbird optimizer (AHA) to obtain a fractional-order PID design for the same benchmark class of regulation systems, highlighting the effectiveness of modern bio-inspired search operators for controller tuning^32^. Beyond regulation benchmarks, grey wolf optimization (GWO) has been used to tune PID parameters for grid-connected PV inverter control, demonstrating that metaheuristic-guided PID tuning can be transferred to power-electronic applications with stringent transient requirements^33^. Moreover, GA-based PID tuning has been integrated with tariff-aware operational objectives in bidirectional EV charging control, indicating that optimization-driven PID design can be extended to multi-objective and cost-constrained control settings^34^. Collectively, these recent results reinforce the growing interest in hybridization and metaheuristic learning mechanisms for PID-type controller tuning, motivating the present study to couple SOO’s oscillatory exploration with DE’s mutation-crossover refinement for high-performance PID-F tuning in nonlinear CSTR temperature regulation.

Recent advances in nature-inspired computation have introduced a new generation of algorithms grounded in astrophysical and biological analogies. The stellar oscillation optimizer (SOO), for instance, models the pulsation behavior of stars observed in asteroseismology to navigate the solution space through oscillatory expansion and contraction phases^35^. This mechanism provides a natural balance between diversification (global exploration) and intensification (local exploitation). However, it is worth noting the original SOO may exhibit reduced convergence speed or precision when the oscillation amplitude decays near local optima, limiting its fine-tuning ability in high-dimensional or stiff nonlinear problems such as CSTR temperature control^36^. These limitations motivate the exploration of hybridization strategies that combine complementary strengths of multiple metaheuristics to achieve a more robust and adaptable optimization process. By embedding secondary operators such as mutation, crossover, or local search phases within primary global exploration algorithms, hybrid methods can dynamically adjust the trade-off between exploration and exploitation throughout the optimization process. DE is well known for its efficient local refinement and strong global convergence properties, provides an ideal candidate for such integration^37^. When coupled with exploration-oriented algorithms, DE has consistently demonstrated improved solution accuracy and stability across engineering design, machine learning, and control applications. Building upon these insights, this study proposes a hybrid SOO with DE (hSOO-DE) for high-performance tuning of PID controllers with derivative filtering (PID-F) in nonlinear CSTR temperature regulation. The key innovation lies in embedding DE’s mutation-crossover operators within the oscillatory search framework of SOO, forming a sequential two-stage optimization cycle. During the early exploration stage, the oscillatory dynamics of SOO enable wide coverage of the parameter space, avoiding premature convergence. In the subsequent exploitation phase, DE refines promising solutions through vector-based recombination, enhancing convergence precision and accelerating the search toward the global optimum. This cooperative hybridization ensures a self-adapting balance between exploration and exploitation, tailored to the evolving needs of the optimization process. In parallel, the controller design in this study employs a PID-F architecture, which extends the conventional PID controller by including a first-order low-pass filter in the derivative term^38^. This configuration mitigates the amplification of measurement noise and reduces the so-called “derivative kick,” thereby improving control smoothness and robustness-critical factors in chemical reactors where measurement signals may contain process noise and temperature fluctuations^39^. The tuning of the four PID-F parameters (proportional, integral, derivative gains, and derivative filter constant) is formulated as a nonlinear optimization problem, where the objective function jointly minimizes overshoot and the integral absolute error. This composite cost ensures simultaneous improvement of transient and steady-state performance.

The remainder of this paper is organized as follows: In Sections “Stellar oscillation optimizer” and “Hybrid stellar oscillation optimizer with differential evolution (hSOO-DE)”, SOO and a new hybrid metaheuristic algorithm (hSOO-DE) is introduced by embedding DE within SOO’s oscillatory dynamics, achieving adaptive cooperation between global exploration and local exploitation, respectively. In Section “Dynamic modeling of continuous stirred tank reactor (CSTR) process”, a benchmark nonlinear CSTR model for controller evaluation is presented; while a robust PID-F controller design framework based on the hSOO-DE is developed for nonlinear CSTR temperature regulation in Section “Proposed novel control method for CSTR process”. In Section “Simulation results and discussion”, the hybrid optimizer is compared with four state-of-the-art metaheuristics (SOO, birds-of-prey-based optimization (BPBO), covariance matrix adaptation evolution strategy (CMA-ES), and DE) as well as classical PID tuning methods including Ziegler-Nichols, Tyreus-Luyben, and Simulink auto-tuner. Performance is assessed through multiple complementary metrics: convergence curves, boxplot and statistical analyses (including Mann-Whitney U-tests), transient response indices (rise time, settling time, overshoot), and integral error measures (IAE, ISE, ITAE, ITSE). A conclusion for this study and the direction for future work is presented in Section “Conclusion and future works”.

## Stellar Oscillation optimizer

### Inspiration and overview

The stellar oscillation optimizer (SOO)^35^ models the expansion-contraction (pulsation) behavior of stars from asteroseismology to steer a population of candidate solutions through exploration (outward, sine-modulated motion) and exploitation (inward, cosine-modulated motion). The algorithm monitors the “luminosity” (fitness) of oscillators over time (an analogue of a light curve) and lets the best oscillators guide the rest of the population, thereby maintaining a principled balance between diversity and convergence.

### Mathematical model

Let ${N}_{osc}$ denote the number of oscillators and $D$ the decision-space dimension. Positions are initialized within $\left[lb,ub\right]\subset{\mathbb{R}}^{D}$ and evaluated by a fitness function $f\left(\cdot\right)$. Initial best luminosity/position are set and updated as the search proceeds^40^.

*Oscillation schedule*: The period $P\left(t\right)$ increases linearly with iteration $t$, the angular frequency is $\omega\left(t\right)=\frac{2\pi}{P\left(t\right)}$ and a scaling term $S\left(t\right)$ decreases linearly with the total iteration budget $T$; these quantities jointly modulate step size and tempo (globally broad early, locally fine later).

1 $$P\left( t \right) = P_{0} + \Delta Pt,\;\;\omega \left( t \right) = \frac{{2\pi }}{{P\left( t \right)}},\;\;S\left( t \right) = 2 - 2\frac{t}{T}$$

These forms (and their exploration-exploitation rationale) are explicitly given in^35^.

*Sine-cosine candidate position*: For oscillator $i$ and component $j$, two trial positions are generated around the current global best ${x}_{best}\left(j\right)$ by sine- and cosine-modulated displacements that depend on $\omega\left(t\right)$, $S\left(t\right)$, random coefficients ${r}_{1}$, ${r}_{2}$, $r_{3} \sim U\left[ {0,1} \right]$ and the current point ${x}_{i}\left(j\right)$^41,42^.

2 $${x}_{i}^{osc1}\left(j\right)={x}_{best}\left(j\right)-{r}_{1}{r}_{3}\left(\omega\left(t\right)S\left(t\right){r}_{1}-S\left(t\right)\right)\left({x}_{i}\left(j\right)-\left|{r}_{1}.\mathrm{sin}\left({r}_{2}\right)\cdot\left|{{r}_{3}x}_{best}\left(j\right)\right|\right|\right)$$

3 $${x}_{i}^{osc2}\left(j\right)={x}_{best}\left(j\right)-{r}_{2}{r}_{3}\left(\omega\left(t\right)S\left(t\right){r}_{1}-S\left(t\right)\right)\left({x}_{i}\left(j\right)-\left|{r}_{1}.\mathrm{cos}\left({r}_{2}\right)\cdot\left|{{r}_{3}x}_{best}\left(j\right)\right|\right|\right)$$

where the term $\left(\omega\left(t\right)S\left(t\right){r}_{1}-S\left(t\right)\right)$ adjusts both direction and magnitude with iteration, while ${r}_{1}$, ${r}_{2}$, ${r}_{3}$ introduce controlled stochasticity.

*Aggregation and probabilistic update*: The two oscillatory positions are aggregated with a random weight (averaging) to form a coordinate-wise candidate, then a probabilistic coordinate update is applied.

4 $${x}_{new}\left(j\right)={r}_{3}\frac{{x}_{i}^{osc1}\left(j\right)+{x}_{i}^{osc2}\left(j\right)}{2}$$

5 $$x_{{new}} \left( j \right) = \left\{ {\begin{array}{*{20}c} {x_{{osc}} \left( j \right),\quad if\;r_{j} \le 0.5} \\ {x_{{old}} \left( j \right),\quad otherwise} \\ \end{array} } \right.$$

where ${x}_{new}\left(j\right)$ denotes the updated coordinate of the $j$-th dimension. At each iteration, the position has a 50% probability of being replaced by the new oscillatory value ${x}_{osc}\left(j\right)$; otherwise, it remains at its previous state ${x}_{old}\left(j\right)$. This stochastic updating mechanism introduces an additional degree of randomness into the search dynamics, enhancing the algorithm’s ability to avoid premature convergence^43^.

The fitness corresponding to each updated position is then evaluated. When the newly obtained fitness value surpasses the current best, the global records are revised as expressed in Eq. (6):

6 $$f\left({x}_{new}\right)<{f}_{best}\Rightarrow{f}_{best}=f\left({x}_{new}\right),\quad{x}_{best}={x}_{new}$$

Here, $f\left({x}_{new}\right)$ denotes the fitness associated with the newly generated position. If this value is superior to the previous ${f}_{best}$, both the best fitness and its corresponding position are replaced by the new ones, ensuring continual improvement of the global solution^44^.

## Hybrid stellar Oscillation optimizer with differential evolution (hSOO-DE)

### Motivation and rationale

Although the SOO^35^ exhibits powerful exploration through its oscillatory dynamics, its convergence speed may slow near local optima when the oscillators’ amplitude decays. To enhance the exploitation strength while maintaining population diversity, the proposed hSOO-DE incorporates the mutation and crossover mechanisms of DE^37^ into the exploitation phase of SOO. DE is particularly known for its efficient local search, stability, and capability to refine promising solutions through vector differentials. By embedding these operators within SOO’s oscillatory cycle, hSOO-DE ensures an adaptive transition from wide exploration to focused exploitation, reducing premature stagnation and accelerating convergence toward the global optimum.

### Differential evolution mechanism

DE is a population-based stochastic optimizer that evolves a set of ${N}_{p}$ candidate vectors ${X}_{i}=\left[{x}_{i,1},{x}_{i,2},\dots.{x}_{i,D}\right]$ over successive generations. The process consists of three primary stages (mutation, crossover, and selection) expressed as follows.

*Mutation*: For each target vector ${X}_{i}^{\left(t\right)}$, a mutant vector ${V}_{i}^{\left(t\right)}$ is generated according to the differential mutation strategy.

7 $${V}_{i}^{\left(t\right)}={X}_{i}^{\left(t\right)}+F\cdot\left({X}_{{r}_{1}}^{\left(t\right)}-{X}_{{r}_{2}}^{\left(t\right)}\right)$$

where ${r}_{1}$,${r}_{2}$ are distinct random indices within $\left[1,{N}_{p}\right]$ and $F\in\left(\mathrm{0,2}\right)$ is the scaling factor controlling the perturbation magnitude.

*Crossover*: The trial vector ${U}_{i}^{\left(t\right)}$ is created by combining components of ${V}_{i}^{\left(t\right)}$ and ${X}_{i}^{\left(t\right)}$ based on the crossover probability${C}_{r}$.

8 $$U_{i}^{{\left( t \right)}} \left( j \right) = \left\{ \begin{gathered} V_{i}^{{\left( t \right)}} \left( j \right),\quad if\;rand_{j} \le C_{r} orj = j_{{rand}} \, \hfill \\ X_{i}^{{\left( t \right)}} \left( j \right),\quad otherwise \hfill \\ \end{gathered} \right.$$

The randomly chosen dimension ${j}_{rand}$ guarantees at least one mutated component.

*Selection*: The fitness of each trial vector is evaluated, and the better between ${U}_{i}^{\left(t\right)}$ and ${X}_{i}^{\left(t\right)}$ is kept for the next generation.

9 $$X_{i}^{{(t + 1)}} \left( j \right) = \left\{ \begin{gathered} U_{i}^{{\left( t \right)}} ,\quad if\;f\left( {U_{i}^{{\left( t \right)}} } \right) \le \left( {X_{i}^{{\left( t \right)}} } \right) \hfill \\ X_{i}^{{\left( t \right)}} ,\quad otherwise \hfill \\ \end{gathered} \right.$$

This greedy selection rule ensures that population quality never degrades over iterations.

### Integration of DE into SOO: the hybrid strategy

In the proposed hybrid framework, the SOO equations (Eqs. 1–6) first update all oscillators to generate sine- and cosine-modulated candidates around the global best ${x}_{best}$. These oscillatory positions act as the initial search basis for the subsequent DE refinement stage. Then, for each oscillator $i$, a subset of these candidates is subjected to DE’s mutation and crossover operations (Eqs. 7–8), producing trial vectors ${U}_{i}$ that exploit the local region discovered by the oscillatory motion. Fitness evaluation and greedy selection (Eq. 9) determine whether each ${U}_{i}$ replaces its parent. This sequential coupling of SOO’s global pulsation dynamics with DE’s differential perturbations enables hSOO-DE to maintain diversity in the early stages and gradually intensify convergence as iterations proceed.

The integrated algorithm operates as follows: after initialization, the population undergoes SOO-based oscillation to explore the global domain; DE mutation and crossover then refine these exploratory moves; fitness-based selection updates the population; and the global best solution is iteratively recorded. The process repeats until the termination criterion (maximum iteration or satisfactory fitness) is met.

**Fig. 1:**
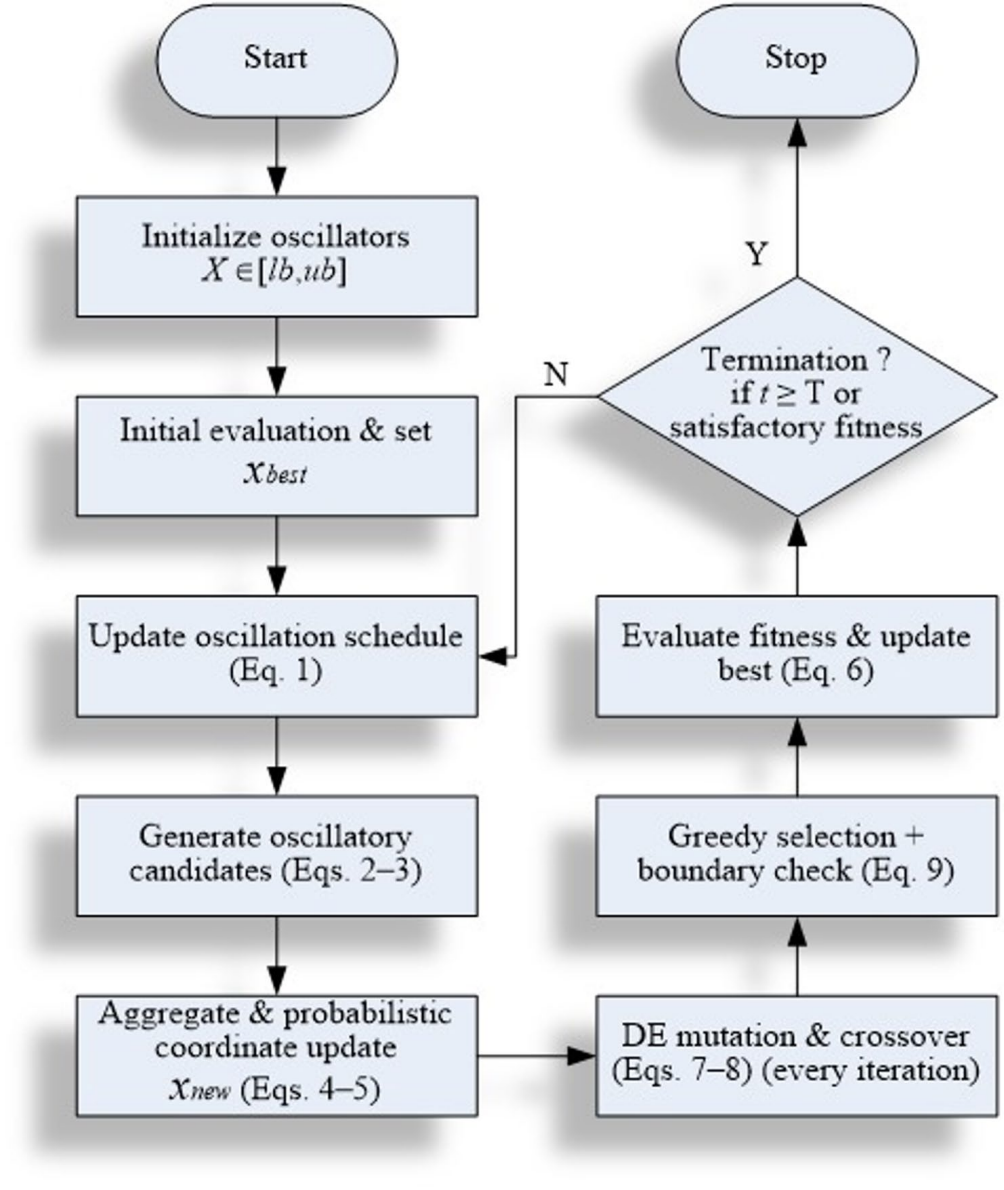
Working mechanism of proposed hSOO-DE algorithm.

To explicitly clarify the integration mechanism, the DE operators are applied sequentially after each SOO-based oscillatory update at every iteration. In this fixed execution order, SOO is responsible for global exploration through its sine-cosine oscillatory dynamics, while DE provides local exploitation by refining the oscillatory candidates via mutation and crossover. No additional switching condition or probabilistic trigger is introduced between the two stages. This deterministic SOO-DE coupling is repeated throughout the optimization process, as illustrated in Fig. 1. The figure conceptually depicts the two-stage search cycle, where oscillators expand and contract around the best-known position according to SOO’s sine-cosine dynamics and DE acts immediately afterward to perturb and recombine these oscillators through vector differencing. The updated trial solutions are then evaluated, and the best-performing individuals are preserved for the next generation. This cyclic interaction between astrophysical oscillation and evolutionary variation allows hSOO-DE to preserve exploration capacity while achieving strong exploitation near the global optimum.

Consequently, the hybridization of SOO with DE combines the astrophysics-inspired oscillatory search behavior with the powerful local refinement of DE. The resulting hSOO-DE offers a dynamic and mathematically transparent framework that improves both convergence stability and optimization accuracy. This algorithmic cooperation is particularly effective for nonlinear, multimodal problems such as reactor temperature control, where exploration of wide parameter spaces must be complemented by precise fine-tuning near the optimal region.

## Dynamic modeling of continuous stirred tank reactor (CSTR) process

**Fig. 2:**
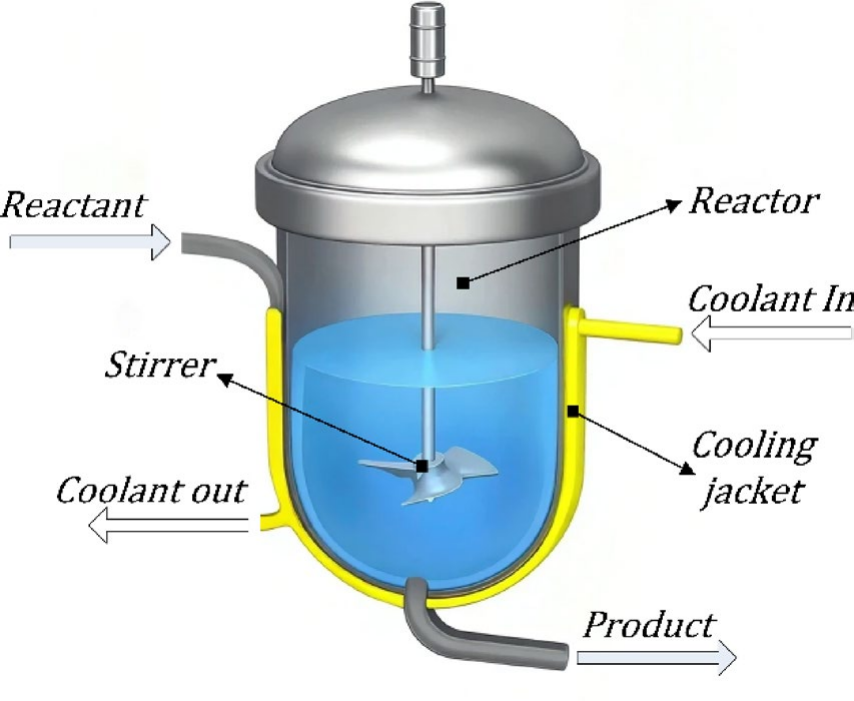
A schematic representation of the jacketed CSTR.

A continuous stirred tank reactor (CSTR) is a canonical benchmark used to analyze nonlinear reaction dynamics and evaluate temperature-control strategies in chemical and process industries. It consists of a continuously stirred vessel into which a liquid feed containing reactant *A* enters at a constant volumetric flow rate ($F$), while the outlet stream leaves at the same rate, ensuring a constant liquid level. The perfect-mixing assumption implies that the outlet composition and temperature are identical to those in the reactor bulk. Because of its strong coupling between reaction kinetics and thermal effects, the CSTR exhibits highly nonlinear behavior and is widely employed to assess the performance of advanced controllers^45–48^. A schematic representation of the jacketed CSTR considered in this work is shown in Fig. 2.

### Reaction description and modeling assumptions

The system performs a single, irreversible, first-order exothermic reaction of the form $A \to B$ in the liquid phase. A cooling jacket surrounds the reactor, through which a heat-exchange medium circulates to remove the heat generated by the reaction. The jacket temperature ${T}_{j}$ acts as the manipulated variable. The following assumptions are adopted in developing the mathematical model^46,48,49^:

I. Perfect mixing ensures uniform temperature and concentration throughout the reactor.
II. The reactor operates at constant liquid volume with equal inflow and outflow rates.
III. Physical properties such as density $\rho$, heat capacity ${c}_{p}$ and heat-transfer coefficient $U$ remain constant.
IV. Gas-phase and pressure effects are neglected.
V. The cooling-jacket temperature ${T}_{j}$ can be manipulated directly, assuming negligible dynamic lag.

### Reaction kinetics

The rate of reaction per unit volume follows the Arrhenius expression:

10 $$r={k}_{0}exp\left(-\frac{E}{RT}\right){C}_{A}$$

where ${k}_{0}$ is the pre-exponential factor ($\mathrm{min}^{-1}$), $E$ is the activation energy (J mol^−1^), $R$ is the universal gas constant (J mol^−1^ K^−1^), $T$ is the reactor temperature ($K$) and ${C}_{A}$ is the concentration of reactant $A$ (mol L^−1^). The exponential temperature dependence introduces strong nonlinearity, making the system highly sensitive to thermal disturbances and parameter variations^45,50^.

### Mass and energy balance

Applying mass and energy conservation principles yields the nonlinear dynamic model of the reactor^46–48^:

11 $$\frac{{dC}_{A}}{dt}=\frac{F}{V}\left({C}_{Af}-{C}_{A}\right)-{k}_{0}{e}^{-E/\left(RT\right)}{C}_{A}$$

12 $$\rho c_{p} \frac{{dT}}{{dt}} = \frac{{F\rho c_{p} }}{V}\left( {T_{f} - T} \right) + \left( { - \Delta H} \right)k_{0} e^{{ - E/\left( {RT} \right)}} C_{A} - \frac{{UA}}{V}\left( {T - T_{j} } \right)$$

where ${C}_{Af}$ and ${T}_{f}$ denote the feed concentration and temperature, respectively; $- \Delta H$ is the heat of reaction (negative for exothermic systems); $U$ and $A$ are the overall heat-transfer coefficient and the heat-exchange area; and ${T}_{j}$ is the coolant temperature. The three terms on the right-hand side of Eq. (12) represent, respectively, convective heat transfer from the feed, heat generation by reaction, and heat removal through the cooling jacket. The strong nonlinear coupling between ${C}_{A}$ and $T$ may lead to multiple steady states and thermal runaway, emphasizing the need for accurate temperature regulation^51^.

### Steady-State conditions and model parameters

At steady state ($\frac{d{C}_{A}}{dt}=0,$ $\frac{dT}{dt}=0$), Eqs. (11)–(12) can be solved simultaneously to determine the equilibrium conditions of the system. The computed steady-state operating points of the reactor are:${T}_{j}=300\,\mathrm{K}$, $T=324.48\,\mathrm{K}$ and ${C}_{A}=0.88\,\mathrm{mol}\,\mathrm{L}^{-1}$. These values are taken as nominal conditions for the subsequent control-system design and optimization studies. The physical and kinetic parameters adopted in this work are summarized in Table 1, corresponding to the standard nonlinear benchmark configuration introduced by^47^.

**Table 1:** Baseline parameters for the CSTR model^45^.

Variable	Value
Activation energy over gas constant ($E/R$)	8750 K
Feed concentration (${C}_{Af}$)	1 mol/L
Heat capacity (${c}_{p}$)	0.239 J/g K
Feed flow rate ($F$)	100 L/min
Reactor volume ($V$)	100 L
Density ($\rho$)	1000 g/L
Feed temperature (${T}_{f}$)	350 K
Heat of reaction ($- \Delta H$)	5 × 10^4^ J/mol
Overall heat-transfer coefficient × area ($UA$)	5 × 10^4^ J/min·K
Pre-exponential factor (${k}_{0}$)	7.2 × 10^10^ min^− 1^

The resulting nonlinear model and parameter set are implemented in MATLAB/Simulink to reproduce the reactor’s dynamic responses and provide the computational framework for the proposed hybrid control scheme.

## Proposed novel control method for CSTR process

### PID with a filter (PID-F)

**Fig. 3:**
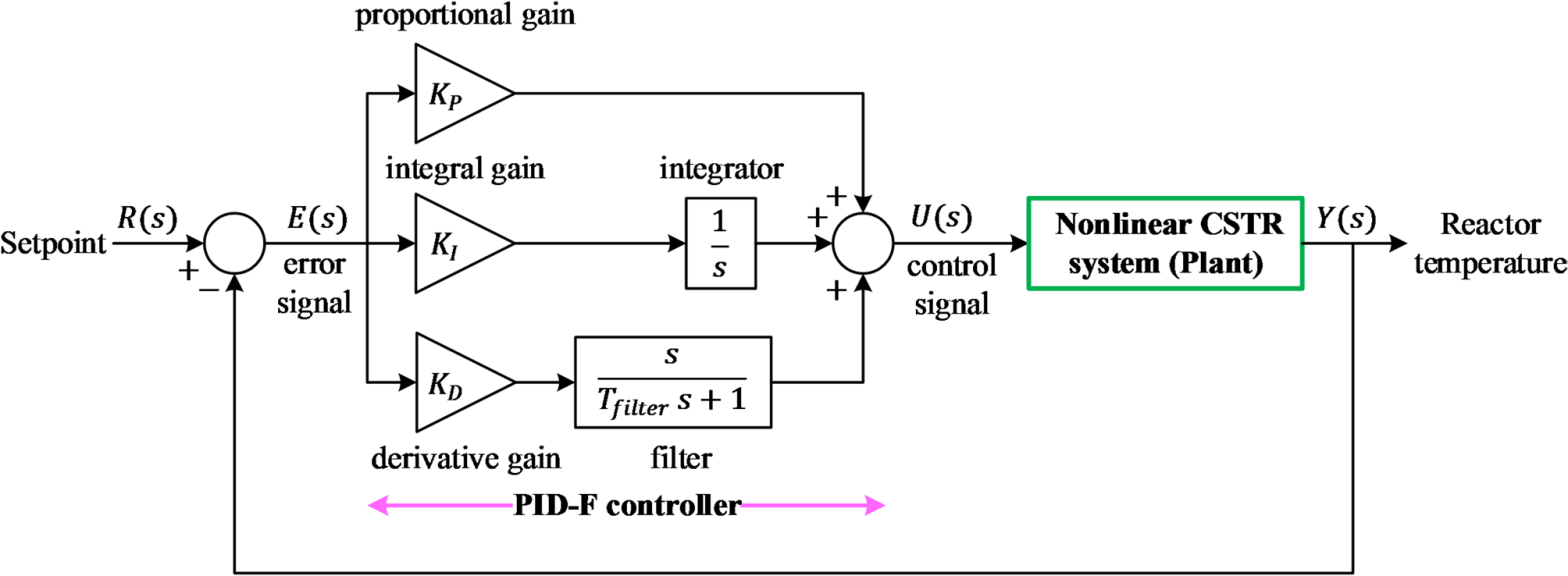
Configuration diagram of the PID-F control scheme.

The classical PID structure was extended with a first-order low-pass filter on its derivative term to enhance robustness against measurement noise and avoid the kick effect commonly associated with sharp reference variations. The resulting controller, denoted as PID-F, is mathematically expressed as:

13 $${PID}_{filter}\left(s\right)={K}_{P}+\frac{{K}_{I}}{s}+{K}_{D}\frac{s}{{T}_{filter}s+1}$$

where ${K}_{P}$ is the proportional gain, ${K}_{I}$ is the integral gain, ${K}_{D}$ is the derivative gain and ${T}_{filter}$ is the derivative-filter time constant. This configuration allows smoother derivative action and improved disturbance rejection when applied to nonlinear thermal processes such as the CSTR. The block diagram of the implemented PID-F control loop is illustrated in Fig. 3, showing how the controller regulates the reactor temperature $T$ through the manipulated jacket-temperature signal. The filter mitigates high-frequency oscillations, ensuring stable control performance even under abrupt load or set-point variations. Similar filtered-derivative configurations have proven effective in suppressing noise amplification and maintaining smooth control in complex industrial systems^52^.

### Objective function and bounds of nonlinear optimization problem

The temperature-regulation task was formulated as a nonlinear optimization problem to identify the optimal PID-F parameters that minimize transient deviations while maintaining rapid convergence to the desired temperature. The CSTR reactor temperature $T$ was subjected to a $20\,\mathrm{K}$ step change in the reference signal from $324.4754\,\mathrm{K}$ to $344.4754\,\mathrm{K}$ at $t=1$ min, allowing the evaluation of both overshoot suppression and settling-time performance.

The minimize objective function was defined as:

14 $$OF=\left(1-\phi\right)\times{PO}_{norm}+\phi\times\underset{0}{\overset{{t}_{sim}}{\int}}\left|e\left(t\right)\right|dt$$

where $e\left(t\right)=r\left(t\right)-y\left(t\right)$ denotes the instantaneous error, ${PO}_{norm}$ is the normalized percentage overshoot, ${t}_{sim}=20\,\mathrm{min}$ is the total simulation duration and $\phi=0.75$ is the weighting factor balancing transient and steady-state accuracy. The integral absolute error ($IAE$) term emphasizes long-term tracking precision, whereas the overshoot term penalizes excessive transient excursions.

The decision variables were the four PID-F parameters ${K}_{P}$,${K}_{I}$,${K}_{D}$ and ${T}_{filter}$. Their lower and upper bounds were defined in this study based on preliminary stability analysis and tuning experience with the nonlinear CSTR model, as no standard ranges are explicitly provided in the literature.

The bounds of PID-F controller: $0.1\le{K}_{P}\le5$, $0.1\le{K}_{I}\le3$, $0.1\le{K}_{D}\le2,0.05\le{T}_{filter}\le0.25$. These limits were selected to avoid actuator saturation while ensuring sufficient control authority over the reactor’s nonlinear thermal response.

### Implementation of proposed hSOO-DE

The hSOO-DE algorithm was developed to identify the optimal PID-F controller parameters for the nonlinear CSTR process. The algorithm combines the exploratory dynamics of the SOO with the robust mutation and crossover strategies of DE.

During the exploration phase, candidate solutions emulate the stellar-motion behavior of SOO, allowing diversified sampling across the search space and reducing the likelihood of premature convergence. In the exploitation phase, DE operators refine the best-performing individuals by generating mutated trial vectors according to^37^:

15 $${X}_{mut}={X}_{r}+F({X}_{p}-{X}_{q})$$

where $F$ is the scaling factor, and ${X}_{p}$, ${X}_{q}$ and ${X}_{r}$ are distinct randomly selected individuals. Crossover and selection operations ensure that only superior offspring progress to the next iteration.

The optimization framework begins with an initial population of PID-F parameter sets constrained within the predefined bounds. Each candidate is evaluated through nonlinear CSTR simulations using the objective function described in Section “Objective function and bounds of nonlinear optimization problem”. The hSOO-DE algorithm iteratively updates the population until the termination criterion (either the maximum number of iterations or negligible improvement in the objective value) is satisfied. The best solution is then implemented as the final controller setting for closed-loop validation.

**Fig. 4:**
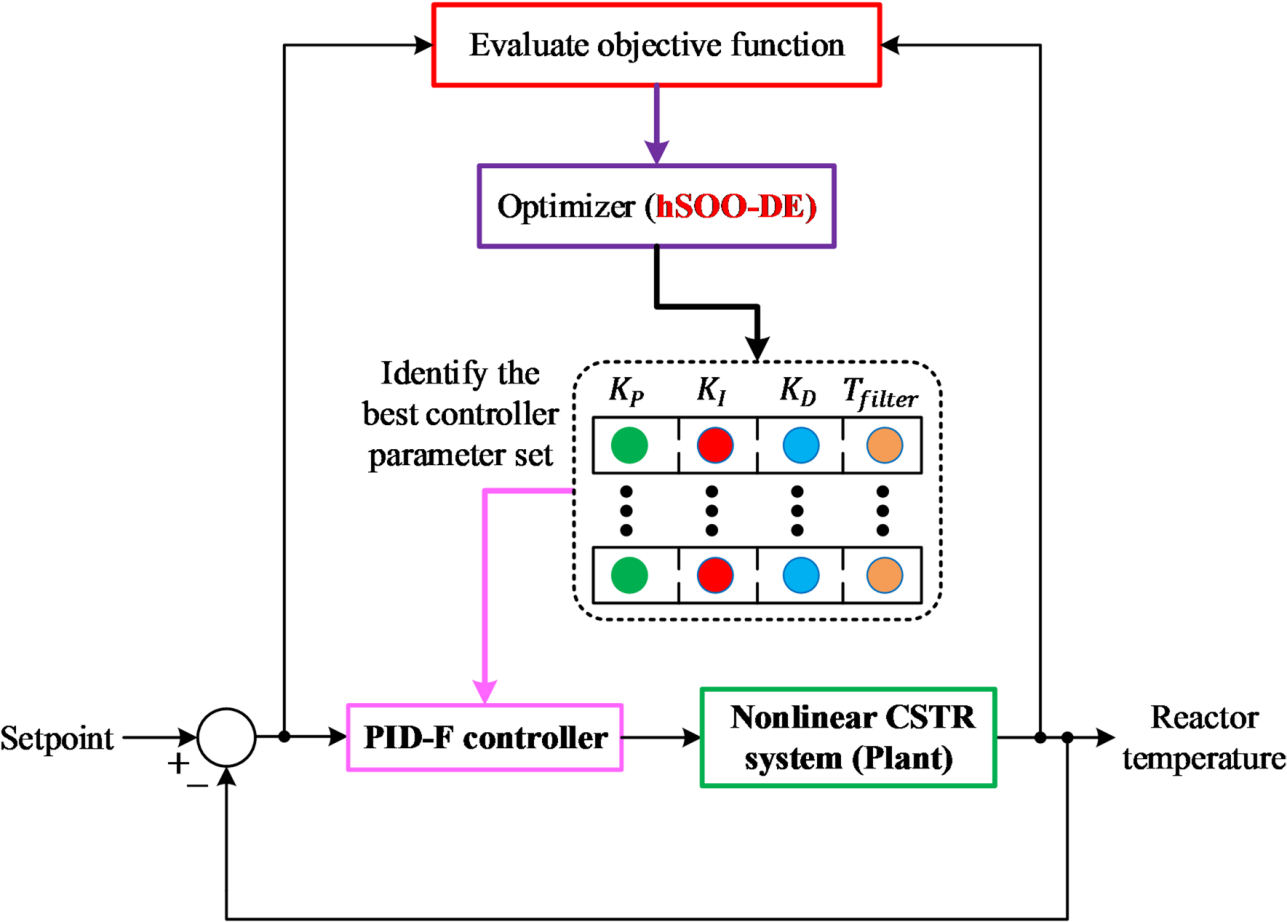
Implementation flow of the hybrid hSOO-DE optimization for PID-F controller tuning in the CSTR system.

## Simulation results and discussion

This section presents a comprehensive evaluation of the proposed hSOO-DE tuned PID-F controller applied to the nonlinear CSTR temperature-regulation problem. The simulations aim to quantitatively assess both the optimization efficiency and the closed-loop control performance of the developed strategy, in comparison with several widely recognized metaheuristic and classical tuning approaches. Five optimizers were employed for benchmarking, namely the proposed hSOO-DE, SOO^35^, BPBO^53^, CMA-ES^54^, and DE^55^, all operating under identical conditions. Each algorithm was executed over 25 independent runs with a population size of 30 and 50 total iterations to ensure statistical reliability.

The comparative investigation proceeds through a sequence of analyses to validate the superiority and robustness of the proposed controller. Section “Boxplot analysis” presents the boxplot and statistical distribution of the obtained objective-function values, while Section “Mann-Whitney U-test” provides a non-parametric Mann-Whitney U-test to confirm the statistical significance of the improvements achieved by hSOO-DE over competing optimizers. Section “Evolution of objective function over iterations and optimal PID-F controller parameters” examines the convergence dynamics and reports the optimal PID-F parameters derived from each algorithm. Section “Time response analysis” evaluates the transient response characteristics of reactor temperature and concentration trajectories, focusing on normalized rise time, settling time, peak time, and overshoot indices. Section “Error-metric analysis” extends this analysis by quantifying error-based performance measures such as normalized steady-state error, IAE, ISE, ITAE, and ITSE.

Subsequently, Section “Comparison with conventionally tuned PID-F controllers” compares the hSOO-DE based controller with conventional PID-F tuning techniques, including Ziegler-Nichols, Tyreus-Luyben, and Simulink auto-tuner methods, to highlight the advancement achieved by the hybrid metaheuristic approach. Finally, Section “Stability performance indicator” employs a modified Zwe-Lee Gaing stability performance indicator to provide an integrated comparison of all optimization and classical strategies in terms of overall stability and dynamic consistency. Through this multi-stage evaluation framework, the section establishes the effectiveness, precision, and robustness of the proposed hybrid algorithm in achieving high-performance temperature regulation in nonlinear CSTR systems.

### Boxplot analysis

**Fig. 5:**
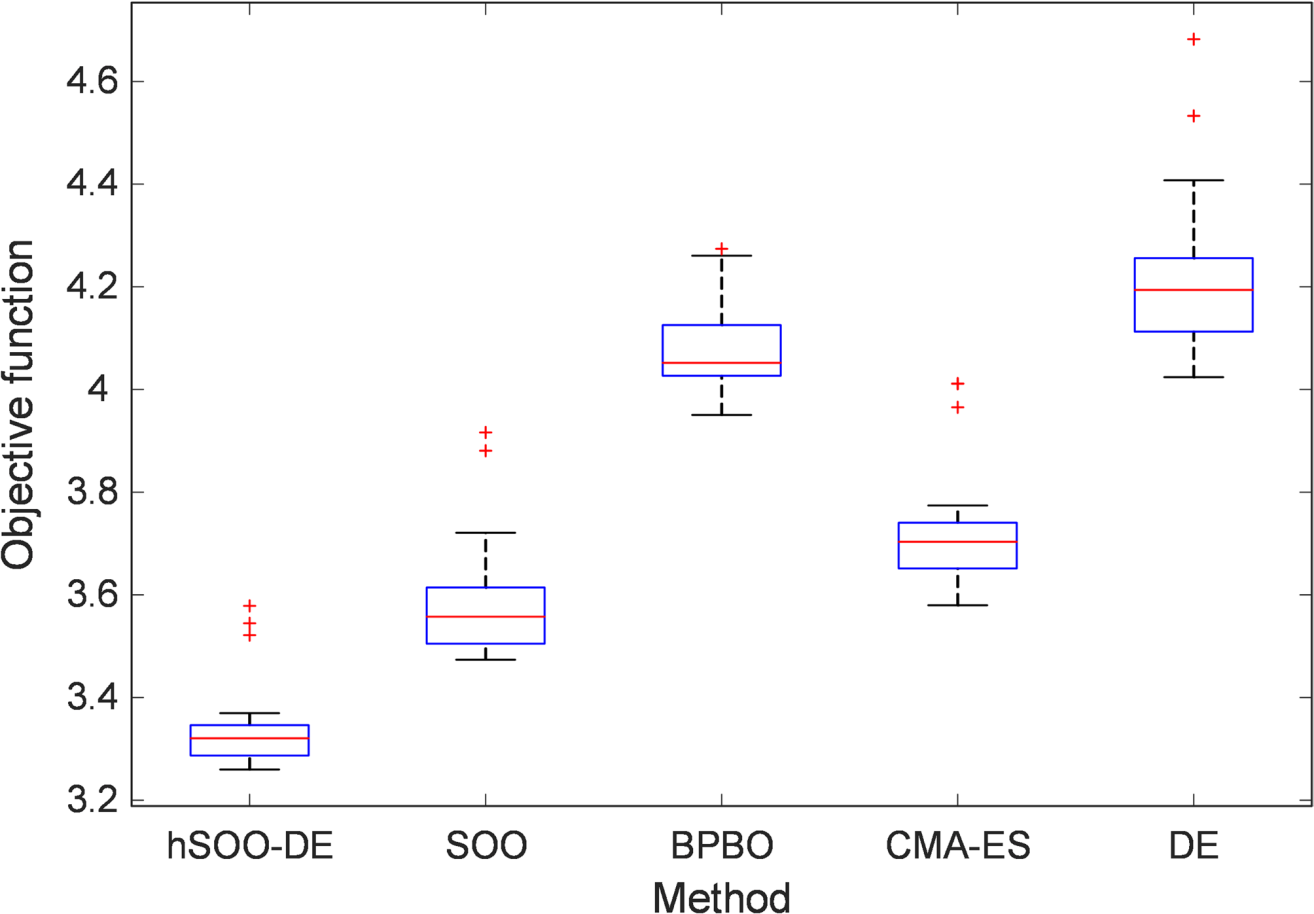
Boxplot analysis of hSOO-DE, SOO, BPBO, CMA-ES and DE.

To examine the statistical robustness and distributional characteristics of the optimization outcomes, the results of 25 independent runs for each algorithm were analyzed using both boxplot visualization and statistical performance metrics. Figure 5 presents the boxplot of the obtained objective-function values for the five comparative optimizers, namely hSOO-DE, SOO, BPBO, CMA-ES, and DE. As illustrated, the proposed hSOO-DE yields the lowest median and the most compact interquartile range (IQR), confirming its superior convergence precision and consistency across multiple trials. In contrast, the original SOO and DE exhibit wider spreads and several outliers, suggesting a higher sensitivity to initial conditions and local stagnation tendencies. The BPBO and CMA-ES algorithms achieve relatively better stability than DE, but their median values remain higher than those of hSOO-DE, indicating slower convergence and less effective local exploitation.

Table 2 summarizes the best, worst, average, and standard-deviation values of the objective function achieved by each optimizer. These metrics collectively quantify the convergence quality and repeatability of each method. The proposed hSOO-DE achieves the smallest average value (3.3385) and the lowest standard deviation (0.0850), which demonstrates its exceptional accuracy and repeatability. By contrast, SOO and DE record higher averages (3.5842 and 4.2135, respectively) and larger dispersions, reflecting weaker balance between exploration and exploitation. The BPBO and CMA-ES methods also produce higher best-case and worst-case values compared with hSOO-DE, confirming that their stochastic search mechanisms cannot attain the same degree of fine-tuning.

**Table 2:** Statistical results of the objective function.

Tuning method	Best	Worst	Average	Standard deviation
hSOO-DE	**3.2598**	**3.5786**	**3.3385**	**0.0850**
SOO	3.4737	3.9162	3.5842	0.1127
BPBO	3.9505	4.2740	4.0831	0.0933
CMA-ES	3.5798	4.0115	3.7200	0.1156
DE	4.0240	4.6823	4.2135	0.1539

The bold values represent the best results.

Overall, both Fig. 5; Table 2 affirm that the integration of DE’s mutation-crossover dynamics within SOO’s oscillatory framework results in an optimizer that not only achieves the lowest objective-function value but also maintains high statistical reliability across independent runs. This robustness establishes hSOO-DE as a consistent and high-precision optimizer suitable for nonlinear control problems such as CSTR temperature regulation.

### Mann-Whitney U-test

To statistically validate the superiority of the proposed hSOO-DE, the non-parametric Mann-Whitney U-test was applied between its final objective-function results and those of the benchmark optimizers (SOO, BPBO, CMA-ES, and DE). This test, which does not assume normality, assesses whether two independent samples originate from the same distribution.

**Table 3:** Comparative Mann-Whitney U-test results.

Metric	hSOO-DE versus SOO	hSOO-DE versus BPBO	hSOO-DE versus CMA-ES	hSOO-DE versus DE
p-value	6.8760E−08	1.4118E−09	1.4118E−09	1.4118E−09
h	1	1	1	1
Winner	hSOO-DE	hSOO-DE	hSOO-DE	hSOO-DE

As shown in Table 3, all p-values are significantly below the 0.05 threshold, indicating that the performance improvements achieved by hSOO-DE are statistically significant. The decision parameter (h = 1) further confirms that the null hypothesis (stating no difference between the compared methods) is rejected in every case. Consequently, hSOO-DE consistently emerges as the statistically dominant optimizer, demonstrating that its hybrid structure yields robust and reproducible convergence behavior across multiple independent runs.

### Evolution of objective function over iterations and optimal PID-F controller parameters

**Fig. 6:**
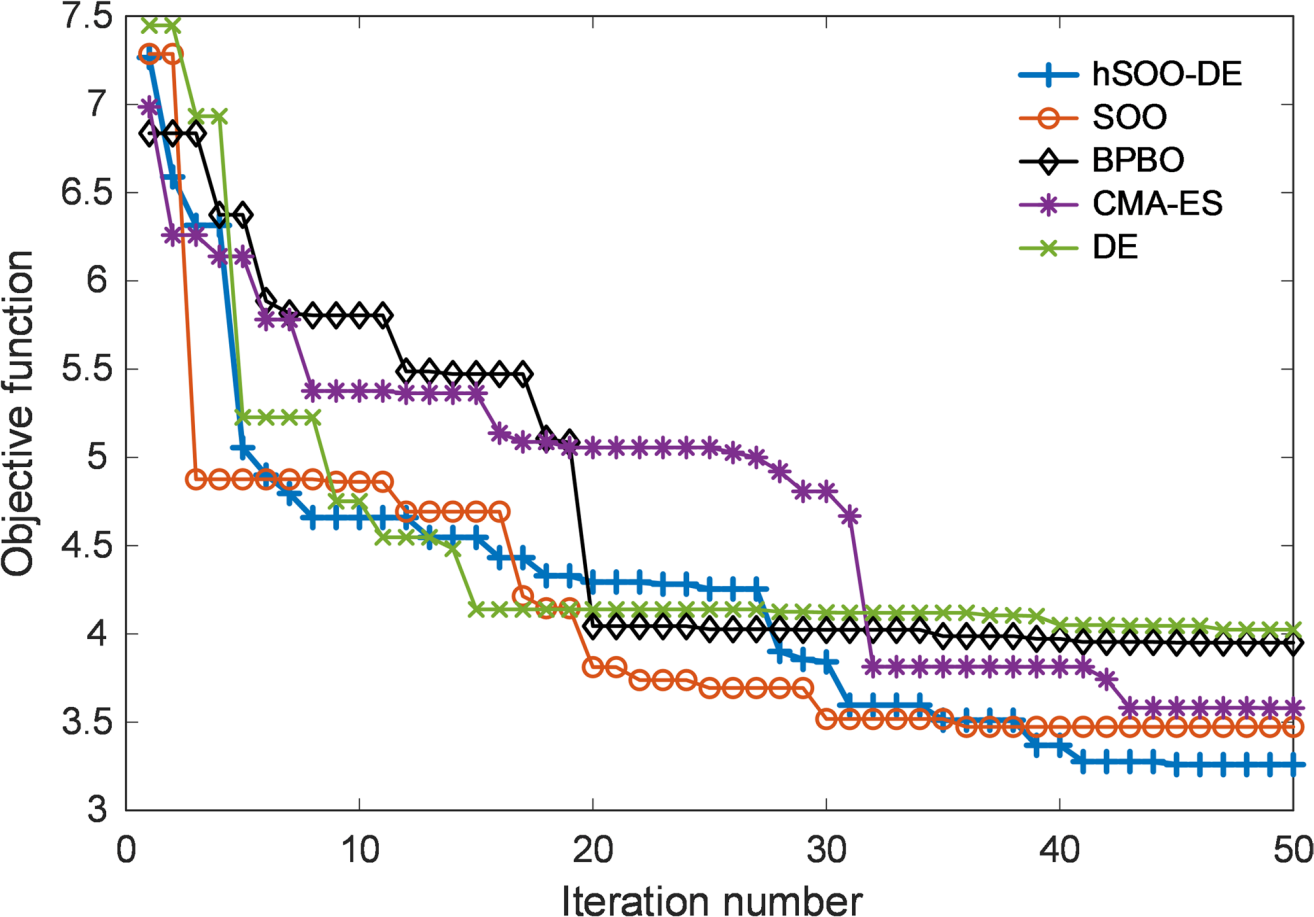
Evolution of objective function for hSOO-DE, SOO, BPBO, CMA-ES and DE.

Figure 6 depicts the convergence trajectories of the objective function for all compared optimizers during the 50-iteration search process. As shown, the proposed hSOO-DE exhibits the fastest and most stable decline, reaching the minimum objective value earlier than the standalone SOO, BPBO, CMA-ES, and DE algorithms. The curve of hSOO-DE demonstrates smooth convergence with limited oscillations, confirming the effectiveness of the hybrid structure in balancing global exploration and local exploitation.

While the original SOO and DE display acceptable convergence patterns, both suffer from mild stagnation in later stages due to limited local refinement. CMA-ES converges relatively steadily but to a higher final cost, and BPBO remains trapped in sub-optimal regions, producing the largest residual objective value among all competitors. These results indicate that integrating DE’s mutation-crossover mechanisms into SOO substantially improves convergence precision and consistency across independent runs.

The optimal PID-F controller gains identified by each method are summarized in Table 4. The proposed hSOO-DE achieved the highest proportional and derivative gains, yielding a faster transient response and superior damping, while maintaining moderate integral and filter terms. This parameter configuration reflects a well-tuned trade-off between tracking speed and stability robustness, ultimately leading to the lowest overall performance index.

**Table 4:** Optimal ${K}_{P}$, ${K}_{I}$, ${K}_{D}$ and ${T}_{filter}$ values obtained by different optimization methods.

Tuning method	${K}_{P}$	${K}_{I}$	${K}_{D}$	${T}_{filter}$
hSOO-DE	1.8520	0.4165	1.9839	0.1811
SOO	1.5517	0.3935	1.8307	0.1746
BPBO	1.5046	0.3517	1.6529	0.1453
CMA-ES	0.7989	0.4307	1.7123	0.2136
DE	1.1364	0.4983	1.7396	0.1732

### Time response analysis

To comprehensively assess the dynamic performance of each tuned controller, the transient and steady-state characteristics of the nonlinear CSTR system were analyzed under a 20 K step increase in reactor temperature setpoint. The relative to the normalized response was defined as:

16 $${y}_{norm}\left(t\right)=\frac{y\left(t\right)-{y}_{initial}}{{y}_{final}-{y}_{initial}}$$

From this normalized curve, the principal temporal indices were computed as follows:

- ${RT}_{norm}$: normalized rise time (10–90% of final value).
- ${ST}_{norm}$: normalized settling time ($\pm2\%$ tolerance band).
- ${PO}_{norm}=\mathrm{max}\left({y}_{norm}\right(t)-1)\times100$: normalized percent overshoot.
- ${PT}_{norm}$: normalized peak time (time at which the peak value occurs).

These indices collectively quantify the speed and damping behavior of each optimized controller.

**Fig. 7:**
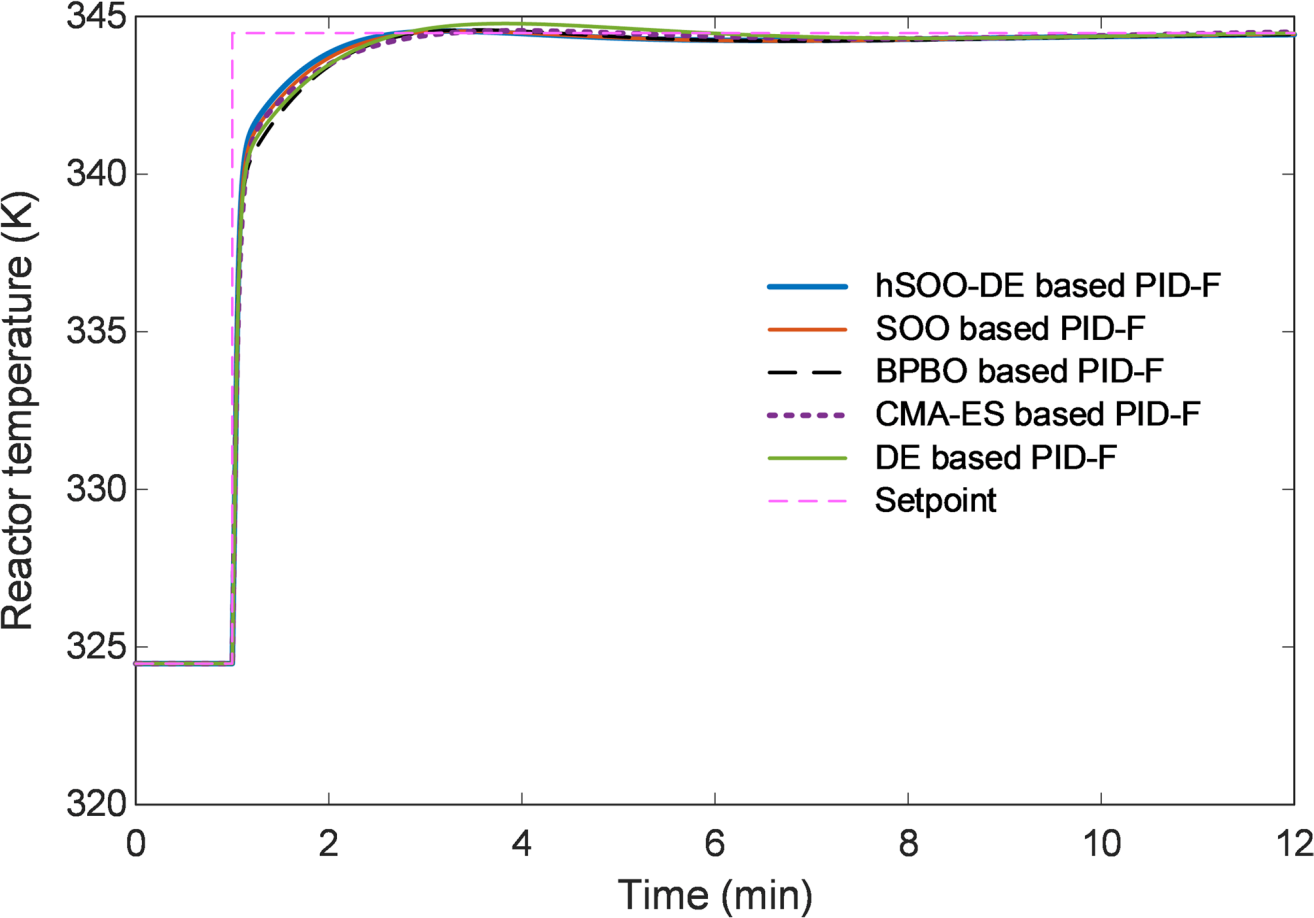
Closed-loop response of reactor temperature for hSOO-DE, SOO, BPBO, CMA-ES and DE tuning methods.

Figure 7 illustrates the closed-loop reactor-temperature responses of the five tuning strategies (hSOO-DE, SOO, BPBO, CMA-ES, and DE). The proposed hSOO-DE achieves the fastest transient with negligible overshoot, settling in approximately 2.17 min, considerably quicker than the other optimizers.

**Fig. 8:**
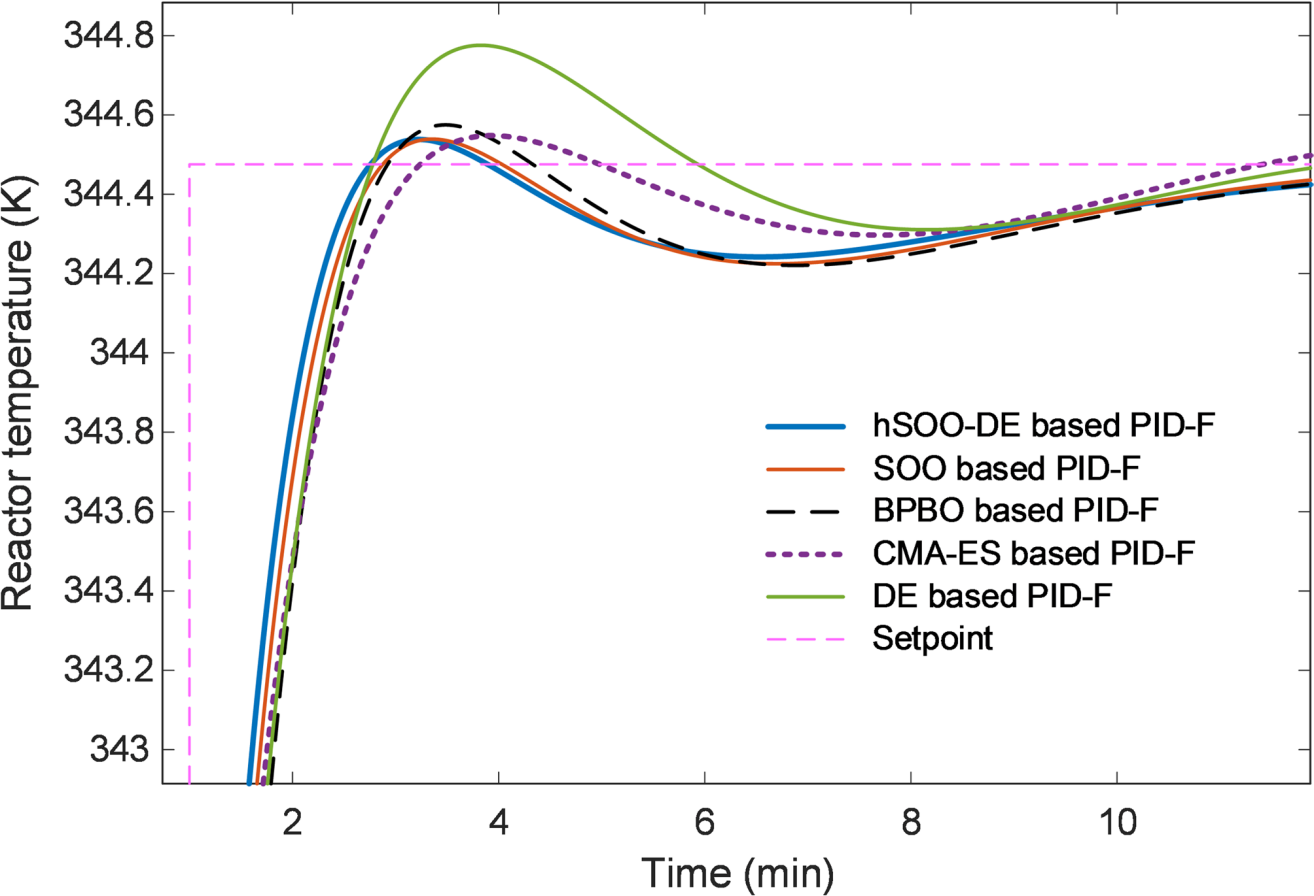
Zoomed view of Fig. 7.

Additionally, the zoomed view in Fig. 8 highlights the superior damping capability of hSOO-DE, which suppresses oscillations more effectively than SOO and DE, while BPBO and CMA-ES exhibit small residual ripples near steady-state.

**Fig. 9:**
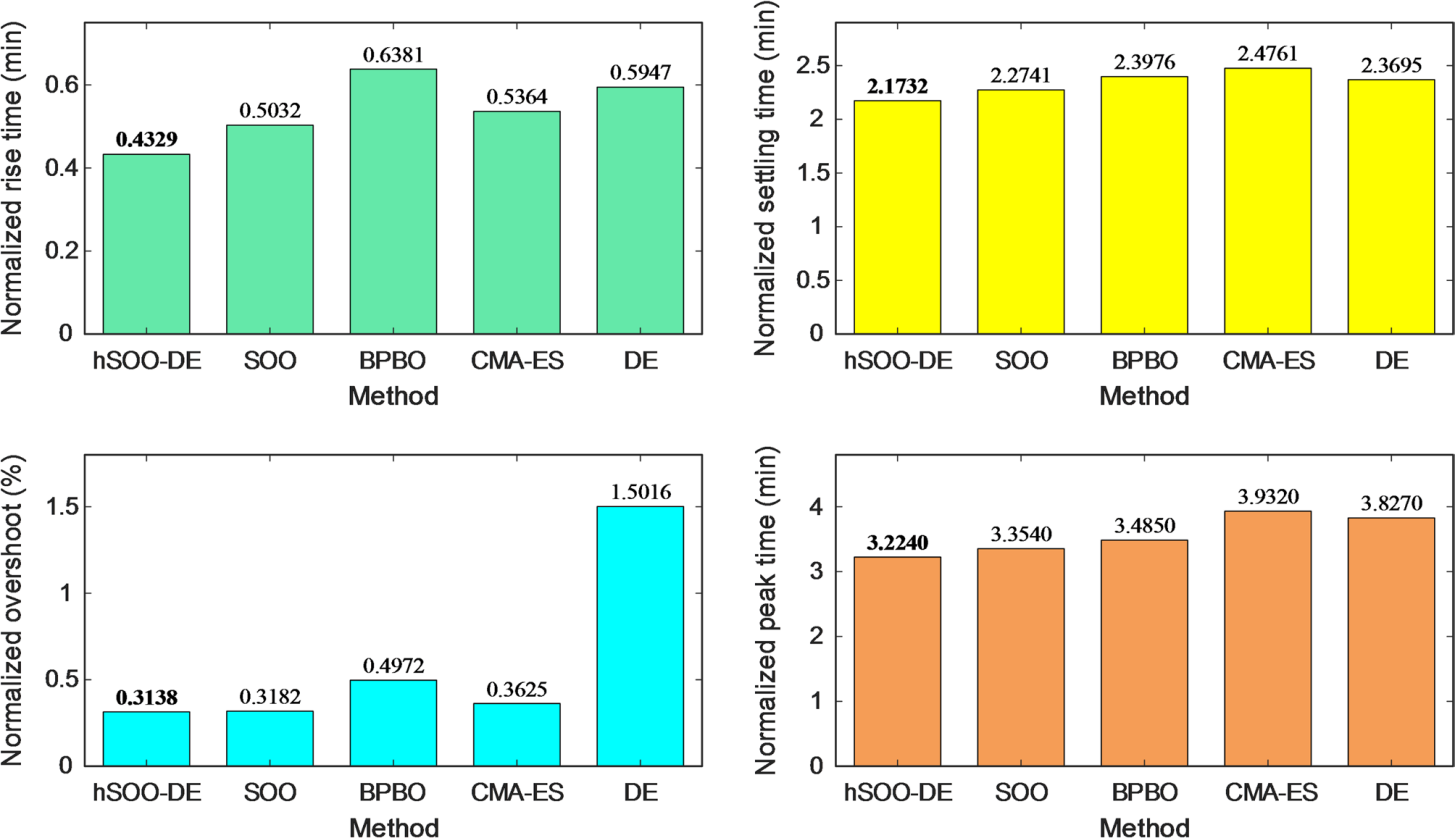
Comparison of normalized stability metrics for hSOO-DE, SOO, BPBO, CMA-ES and DE tuning methods.

The quantitative comparison of normalized transient metrics is presented in Fig. 9, confirming the statistical dominance of hSOO-DE. Specifically, it yields the smallest rise time (0.4329 min), overshoot (0.3138%), and settling time (2.1732 min), while maintaining the lowest peak-time index among all competitors. These results clearly indicate that the hybridized search mechanism enables both rapid tracking and enhanced damping.

**Fig. 10:**
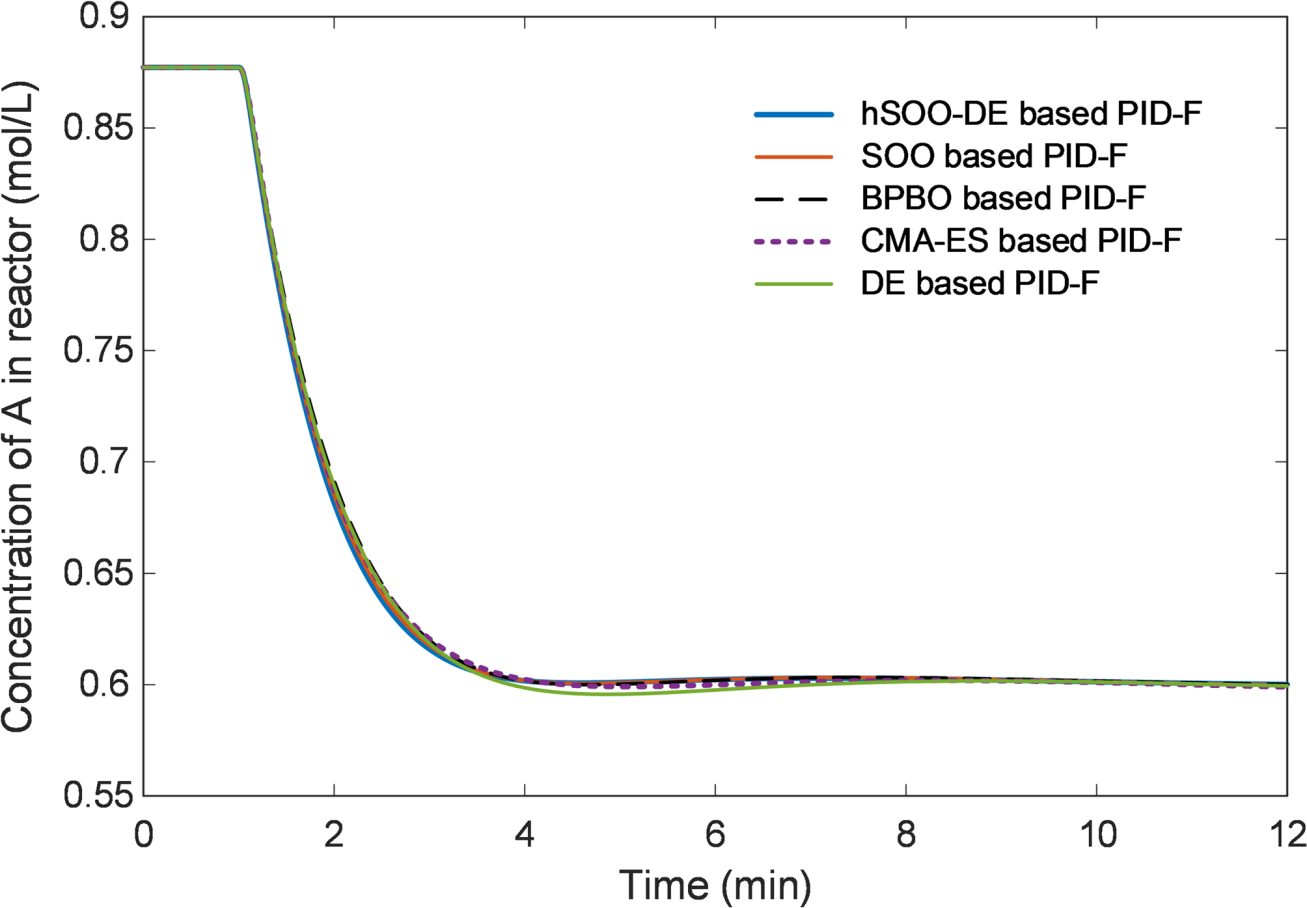
Closed-loop response of concentration of A in reactor (${C}_{A}$) for hSOO-DE, SOO, BPBO, CMA-ES and DE tuning methods.

Finally, Fig. 10 depicts the closed-loop evolution of ${C}_{A}$, confirming that all optimizers maintain chemical stability; however, the hSOO-DE controller yields a faster and smoother concentration transition, confirming its improved regulation capability for strongly coupled reactor variables.

### Error-metric analysis

To further assess quantitative control accuracy, five widely used error-based performance (${SSE}_{norm}$= normalized percent steady-state error, ${F}_{IAE}$= integral of absolute error, ${F}_{ISE}$=integral of squared error, ${F}_{ITAE}$=integral of time-weighted absolute error and ${F}_{ITSE}$=integral of time-weighted squared error) indices were evaluated for all optimization methods. These indices reflect the system’s transient and steady-state performance over the entire simulation horizon (${t}_{sim}=20$ min). The normalized percent steady-state error ( ${SSE}_{norm}$) and four integral-based metrics were calculated as follows:

17 $${SSE}_{norm}=\left|{y}_{norm}\left({t}_{sim}\right)-1\right|\times100$$

18 $$F_{{IAE}} = \int\limits_{0}^{{t_{{sim}} }} {\left| {e\left( t \right)} \right|dt}$$

19 $${F}_{ISE}=\int\limits_{0}^{{t}_{sim}}{e}^{2}\left(t\right)dt$$

20 $${F}_{ITAE}=\int\limits_{0}^{{t}_{sim}}t\left|e\left(t\right)\right|dt$$

21 $${F}_{ITSE}=\int\limits_{0}^{{t}_{sim}}t{e}^{2}\left(t\right)dt$$

Table 5 summarizes the values obtained by each optimizer. The proposed hSOO-DE achieves the lowest score for every metric demonstrating reduced steady-state deviation and smoother transients compared with SOO, BPBO, CMA-ES, and DE. These results corroborate the improvements already observed in the time-response analysis.

**Table 5:** Comparative error metrics for recent tuning methods.

Tuning method	${SSE}_{norm}$	${F}_{IAE}$	${F}_{ISE}$	${F}_{ITAE}$	${F}_{ITSE}$
hSOO-DE	**4.9542E−04**	**4.2418**	**17.8873**	**14.2487**	**20.9180**
SOO	0.0090	4.5256	18.9983	14.5092	22.5592
BPBO	0.0039	5.1015	21.2055	15.9216	25.8005
CMA-ES	0.1234	4.6523	20.3431	15.3833	23.7884
DE	0.0335	4.8648	20.4121	14.5186	24.1470

The bold values represent the best results.

### Comparison with conventionally tuned PID-F controllers

To further validate the effectiveness of the proposed hSOO-DE-tuned PID-F controller, its performance was compared with three conventional tuning techniques: Ziegler-Nichols, Tyreus-Luyben, and the Simulink PID-F tuner. The parameter settings obtained from these classical methods are summarized in Table 6, where notable deviations can be observed in both proportional and derivative gains compared with the optimized hSOO-DE configuration.

**Table 6:** Parameter values of classical PID-F tuning methods.

Tuning method	${K}_{P}$	${K}_{I}$	${K}_{D}$	${T}_{filter}$
Ziegler-Nichols	1.1094	0.4894	0.6287	0.1
Tyreus-Luyben	1.8830	0.3220	0.8416	0.1
Simulink Tuner	1.3831	0.2453	1.1102	0.0934

**Fig. 11:**
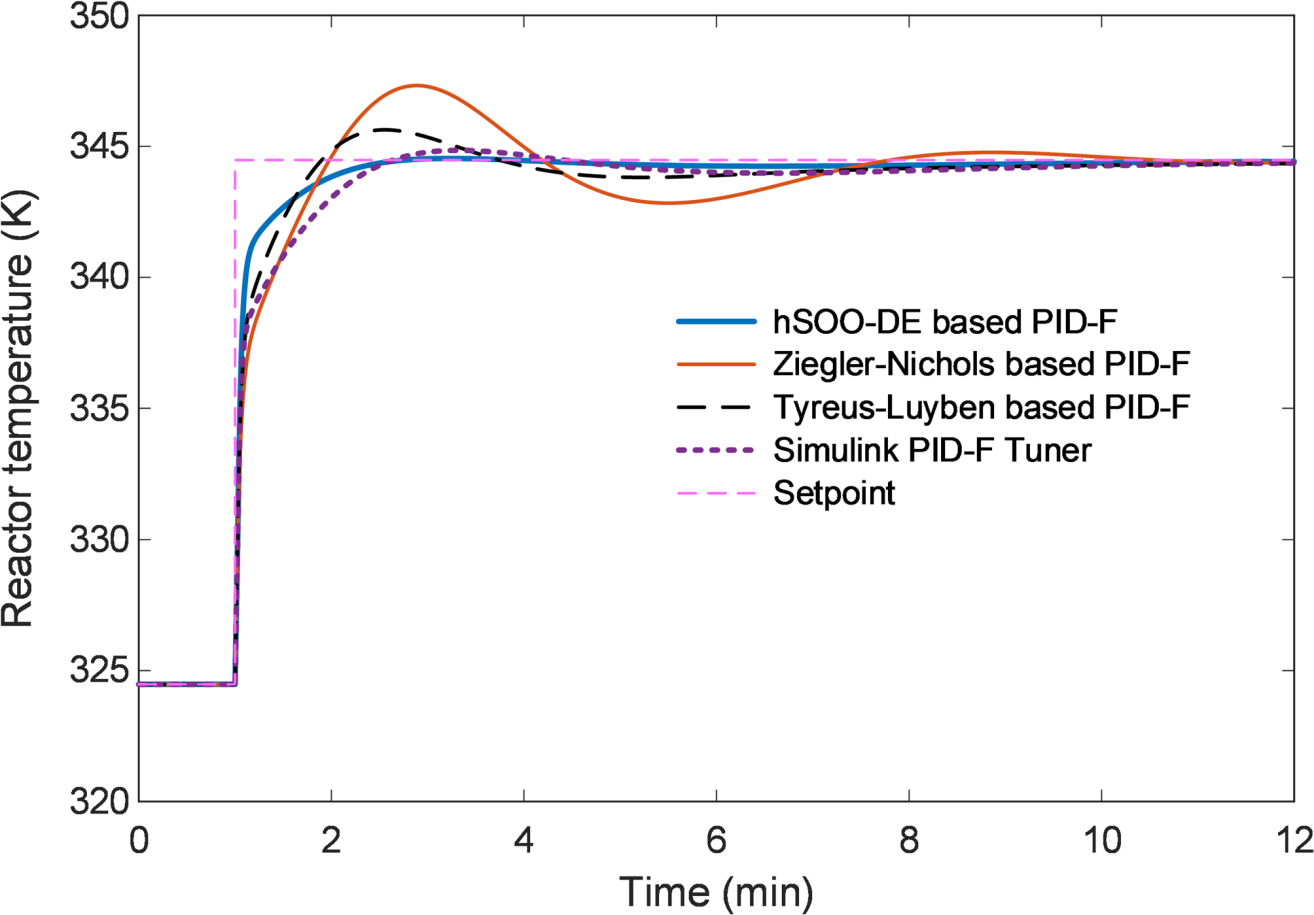
Closed-loop response of reactor temperature for hSOO-DE and classical tuning methods.

The closed-loop temperature responses shown in Fig. 11 reveal that the hSOO-DE-based controller exhibits the fastest rise and settling times with the lowest overshoot, achieving smooth convergence to the desired setpoint. In contrast, the Ziegler-Nichols-tuned controller produces a pronounced transient peak, while the Tyreus-Luyben and Simulink-tuned designs demonstrate slower settling and moderate oscillatory tendencies.

**Fig. 12:**
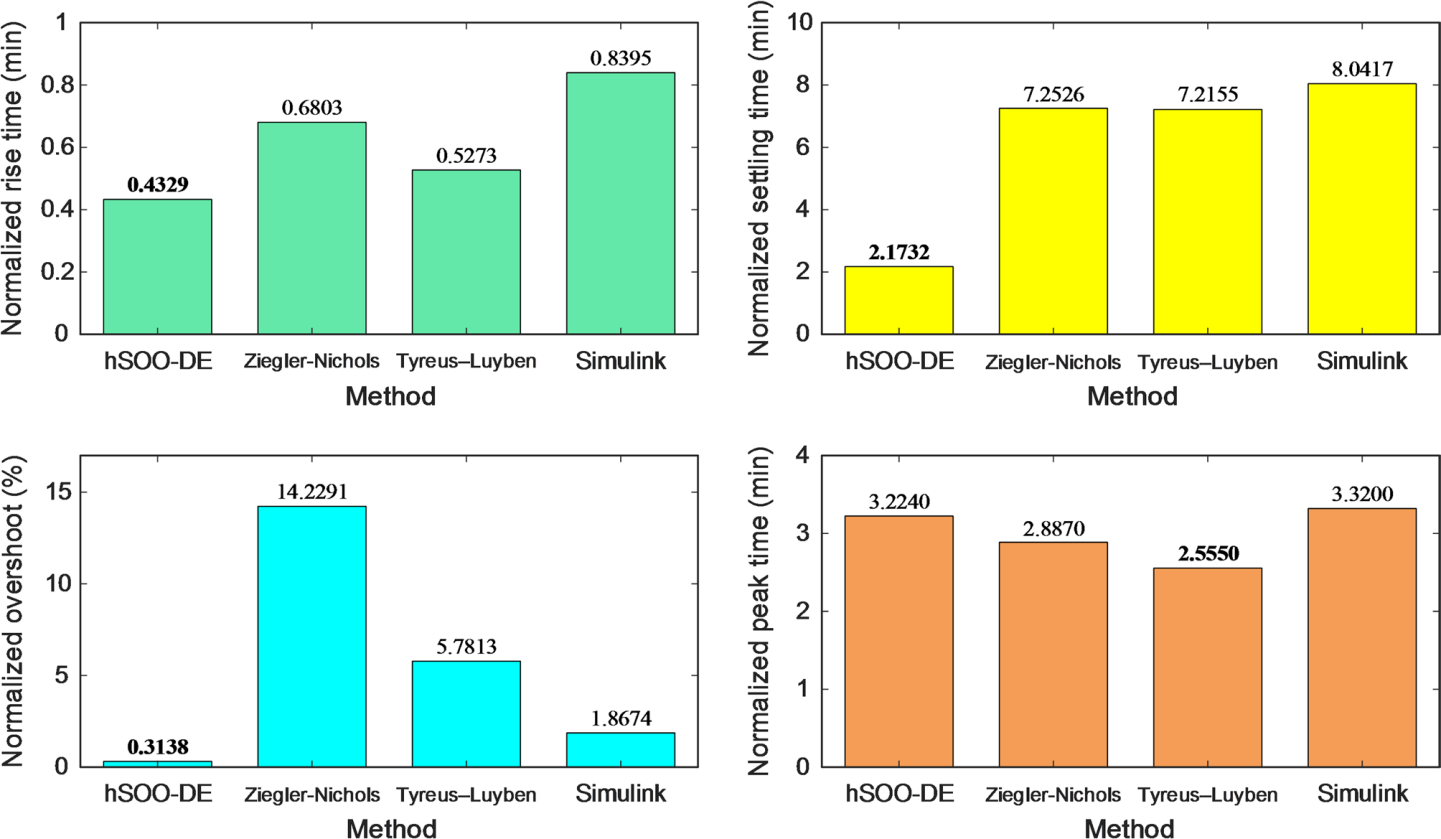
Comparison of normalized stability metrics for hSOO-DE, Ziegler-Nichols, Tyreus-Luyben and Simulink tuner based PID-F controlled systems.

The corresponding normalized stability metrics in Fig. 12 further confirm these observations. The hSOO-DE-based controller yields the smallest rise time, settling time, and overshoot values among all compared methods, demonstrating faster transient behavior and stronger damping characteristics. In contrast, all conventional tuning techniques exhibit noticeably higher stability indices, indicating slower system response and greater oscillatory tendencies.

**Fig. 13:**
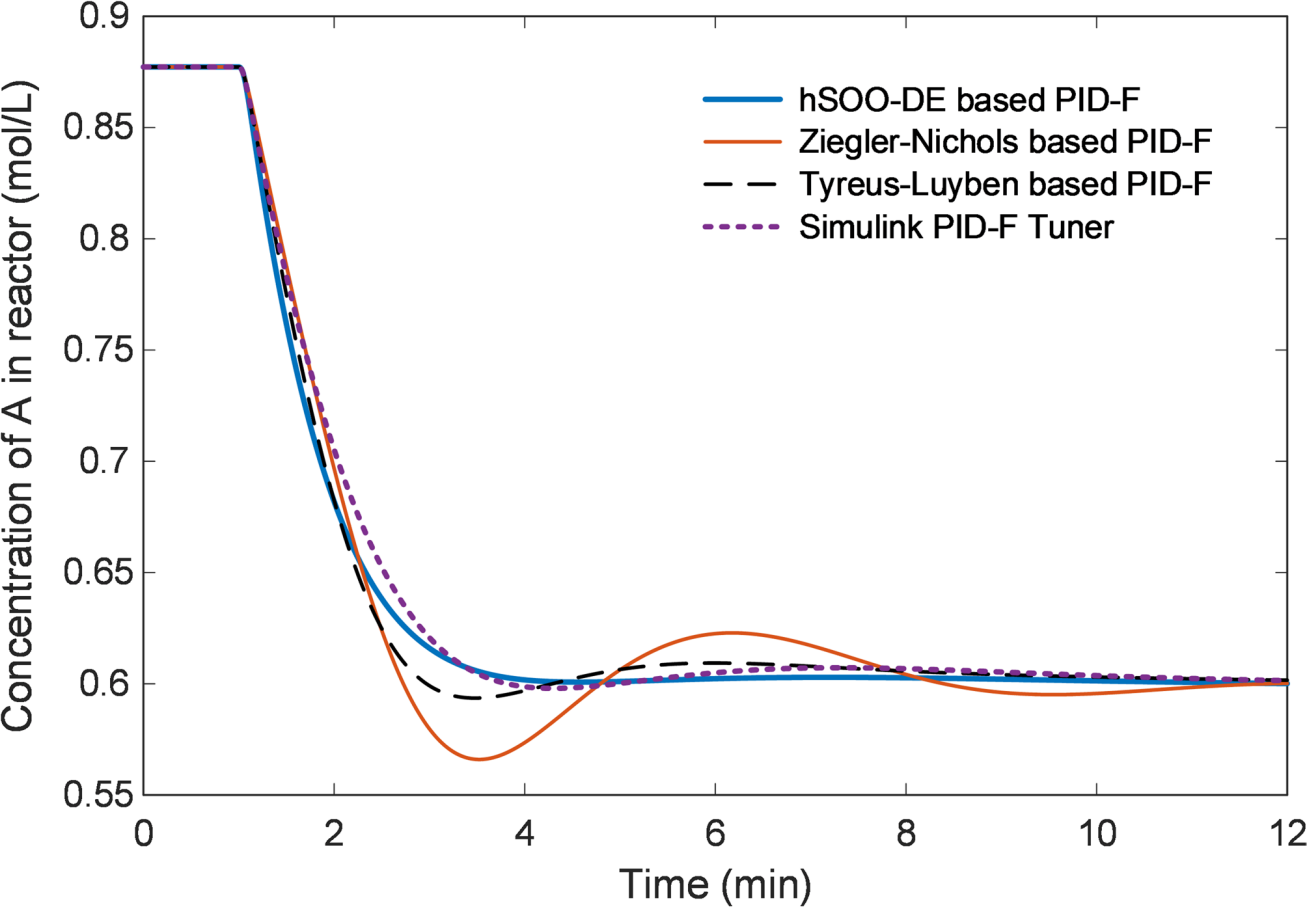
Closed-loop response of concentration of A in reactor (${C}_{A}$) for hSOO-DE and classical tuning methods.

The reactant concentration profiles illustrated in Fig. 13 further emphasize the hybrid optimizer’s advantage. hSOO-DE ensures a smoother decay trajectory of the reactant concentration within the reactor and minimal deviation around the steady-state value, indicating effective disturbance rejection and improved nonlinear stability. Ziegler-Nichols exhibits a pronounced overshoot in concentration, whereas Simulink- and Tyreus-Luyben-tuned controllers converge more slowly.

Finally, the error-metric comparison presented in Table 7 quantitatively substantiates these observations. The proposed hSOO-DE achieves the lowest normalized steady-state error as well as the smallest IAE, ISE, ITAE and ITSE values among compared classical tuning methods. These results clearly demonstrate that the hybrid optimizer provides both superior accuracy and robustness, outperforming conventional PID-F tuning strategies in speed, precision, and error minimization.

**Table 7:** Comparative error metrics for classical tuning methods.

Tuning method	${SSE}_{norm}$	${F}_{IAE}$	${F}_{ISE}$	${F}_{ITAE}$	${F}_{ITSE}$
hSOO-DE	**4.9542E−04**	**4.2418**	**17.8873**	**14.2487**	**20.9180**
Ziegler-Nichols	0.0021	12.4514	44.2130	45.3981	88.0914
Tyreus-Luyben	0.1148	7.8857	25.3111	34.9145	36.9232
Simulink Tuner	0.0611	7.8476	29.3973	31.3279	41.0301

The bold values represent the best results.

### Stability performance indicator

To provide an overall quantitative measure of closed-loop robustness and transient quality, a modified version of the Zwe-Lee Gaing performance indicator$\left({F}_{mZLG}\right)$ was adopted. This metric integrates steady-state and transient characteristics into a single expression, formulated as:

22 $${F}_{mZLG}=\frac{\left(1-{e}^{-\sigma}\right)}{100}\left({PO}_{norm}+{SSE}_{norm}\right)+{e}^{-\sigma}({ST}_{norm}-{RT}_{norm})$$

where ${PO}_{norm}$, ${SSE}_{norm}$, ${ST}_{norm}$ and ${RT}_{norm}$ denote the normalized overshoot, steady-state error, settling time, and rise time, respectively. The stability factor was fixed at $\sigma=1$, ensuring equal weighting between transient and steady-state effects. Lower ${F}_{mZLG}$ values indicate improved damping, faster settling, and smaller deviations from the reference, thus reflecting a superior compromise between responsiveness and stability.

**Fig. 14:**
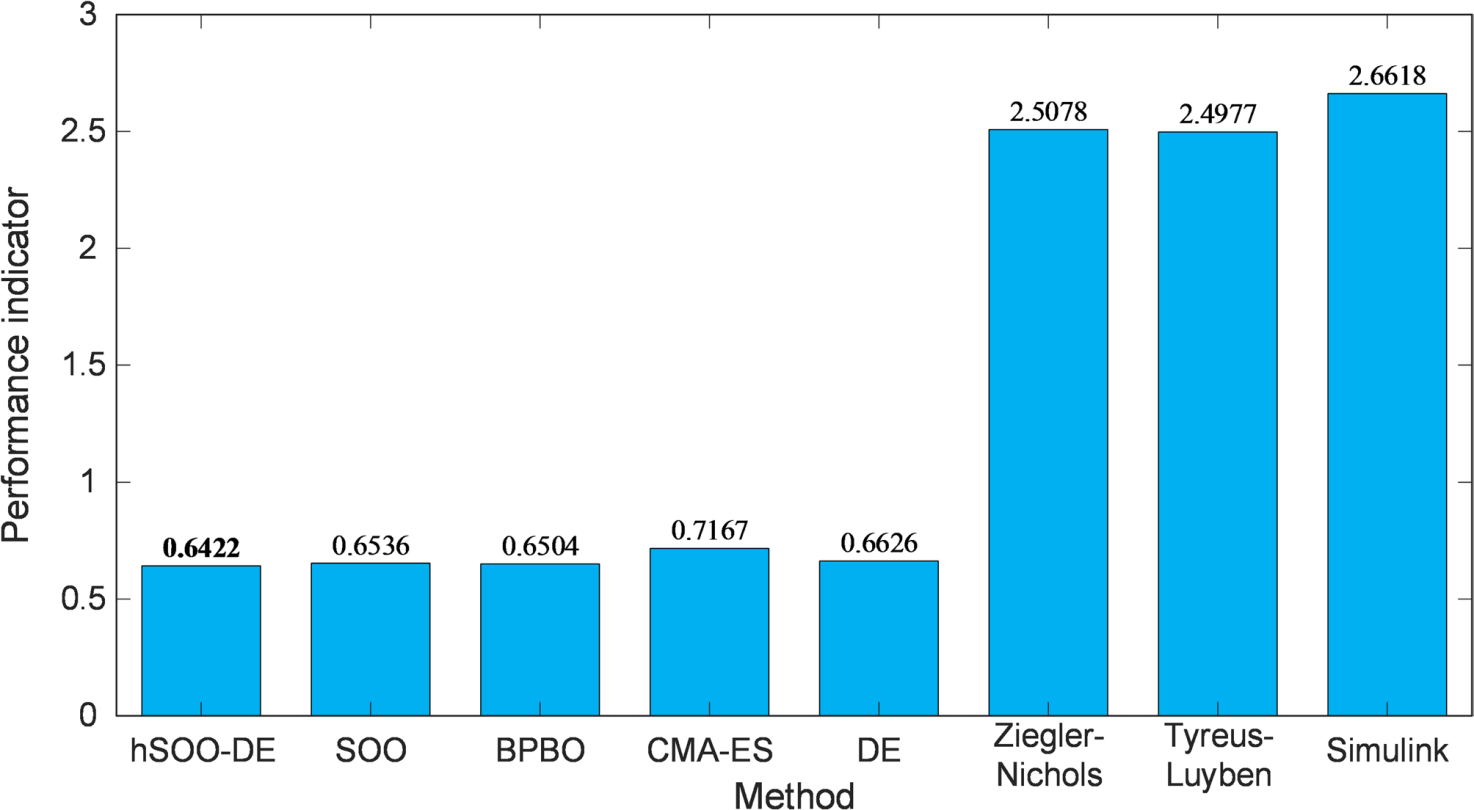
Bar plot of performance indicator ${F}_{mZLG}$.

The comparative results presented in Fig. 14 demonstrate that the proposed hSOO-DE yields the smallest ${F}_{mZLG}$ value among all evaluated methods. Both conventional approaches (Ziegler-Nichols, Tyreus-Luyben and Simulink Tuner) and recent metaheuristics (SOO, BPBO, CMA-ES, and DE) exhibit noticeably higher indicators, confirming less favorable stability margins. This outcome consolidates the overall findings of Sections “Time response analysis–Comparison with conventionally tuned PID-F controllers”, verifying that the hybridization of SOO with DE not only enhances transient behavior but also ensures global stability robustness under nonlinear operating conditions.

### Comparative merits and demerits of tuning methods

To facilitate a consolidated comparison for readers, the relative merits and limitations of the considered tuning approaches are summarized based on the simulation results presented in Section “Simulation results and discussion”. Classical tuning rules such as Ziegler-Nichols and Tyreus-Luyben offer rapid parameter determination with minimal computational effort; however, they exhibit less favorable transient characteristics, including larger overshoot and longer settling behavior under nonlinear operating conditions. The Simulink auto-tuner improves ease of implementation and automation, yet its closed-loop performance remains inferior to metaheuristic-based tuning approaches in terms of normalized transient and error metrics.

Among population-based optimizers, SOO and DE demonstrate balanced performance with acceptable convergence behavior; however, their improvement rate in later iterations is slower when compared with the proposed hybrid approach. BPBO shows global search capability but exhibits higher variability across independent runs, as reflected in the statistical dispersion of the objective function values. CMA-ES achieves stable convergence trends; nevertheless, it converges to a comparatively higher objective value within the adopted iteration budget.

In contrast, the proposed hSOO-DE consistently achieves lower objective-function values, improved convergence behavior, and superior transient-response characteristics across all evaluated performance indices. By integrating the oscillatory exploration mechanism of SOO with the differential mutation-crossover refinement of DE, the hybrid strategy effectively mitigates the individual shortcomings of its constituent algorithms while preserving their complementary strengths. These results indicate that hSOO-DE provides a more balanced and reliable tuning framework for nonlinear CSTR temperature control within the investigated simulation setting.

## Conclusion and future works

A hybrid metaheuristic optimization strategy, hSOO-DE, was developed for tuning PID-F controllers applied to CSTR systems. The algorithm integrates the oscillatory search dynamics of the SOO with the mutation-crossover refinement of DE, achieving a coherent balance between exploration and exploitation. Simulation studies on the benchmark CSTR model demonstrated that the hybrid optimizer significantly improves both convergence efficiency and control precision.

Comparative analyses with state-of-the-art metaheuristic algorithms and classical PID-F tuning methods revealed consistent superiority of hSOO-DE in transient and steady-state performance metrics. The proposed controller yielded smaller overshoot, faster settling time, and minimal steady-state error while maintaining robustness under nonlinear process dynamics. Statistical evaluations, including boxplot and Mann-Whitney U-tests, confirmed that the improvements are not only quantitative but also statistically significant. These findings highlight the capability of the hybrid optimizer to provide an accurate, stable, and computationally efficient tuning framework for nonlinear process control.

Despite the encouraging results obtained in this study, several limitations should be acknowledged. First, the performance evaluation of the proposed hSOO-DE-tuned PID-F controller is conducted entirely through numerical simulations using a benchmark nonlinear CSTR model. Although this model is widely adopted in the literature, experimental validation or real-time implementation has not been considered in the present work. Second, the controller parameters are optimized for a fixed operating condition, and the effects of large parametric uncertainties, unmodeled dynamics, or severe measurement noise have not been explicitly investigated. In addition, the computational complexity associated with hybrid metaheuristic optimization may become more significant for large-scale or real-time control applications. These limitations provide important directions for future research and do not detract from the comparative insights presented in this study.

Future research may extend this framework toward real-time experimental validation on pilot-scale reactors, robust tuning under parameter uncertainty, and adaptive on-line implementations. Expanding the hybrid concept to multi-objective or fractional-order controller architectures could further enhance control flexibility and industrial applicability.

## Data Availability

The datasets generated and/or analyzed during the current study are available from the corresponding author on reasonable request.
